# Customizable, reconfigurable, and anatomically coordinated large-area, high-density electromyography from drawn-on-skin electrode arrays

**DOI:** 10.1093/pnasnexus/pgac291

**Published:** 2023-01-11

**Authors:** Faheem Ershad, Michael Houston, Shubham Patel, Luis Contreras, Bikram Koirala, Yuntao Lu, Zhoulyu Rao, Yang Liu, Nicholas Dias, Arturo Haces-Garcia, Weihang Zhu, Yingchun Zhang, Cunjiang Yu

**Affiliations:** Department of Biomedical Engineering, Pennsylvania State University, University Park, PA, 16801, USA; Department of Biomedical Engineering, University of Houston, Houston, TX, 77204, USA; Department of Biomedical Engineering, University of Houston, Houston, TX, 77204, USA; Department of Engineering Science and Mechanics, Pennsylvania State University, University Park, PA, 16801, USA; Department of Mechanical Engineering, University of Houston, Houston, TX, 77204, USA; Department of Biomedical Engineering, University of Houston, Houston, TX, 77204, USA; Department of Mechanical Engineering, University of Houston, Houston, TX, 77204, USA; Department of Engineering Technology, University of Houston, Houston, TX, 77204, USA; Department of Engineering Science and Mechanics, Pennsylvania State University, University Park, PA, 16801, USA; Materials Science and Engineering Program, University of Houston, Houston, TX, 77204, USA; Department of Engineering Science and Mechanics, Pennsylvania State University, University Park, PA, 16801, USA; Materials Science and Engineering Program, University of Houston, Houston, TX, 77204, USA; Department of Biomedical Engineering, University of Houston, Houston, TX, 77204, USA; Department of Biomedical Engineering, University of Houston, Houston, TX, 77204, USA; Department of Engineering Technology, University of Houston, Houston, TX, 77204, USA; Department of Electrical and Computer Engineering, University of Houston, Houston, TX, 77204, USA; Department of Mechanical Engineering, University of Houston, Houston, TX, 77204, USA; Department of Engineering Technology, University of Houston, Houston, TX, 77204, USA; Department of Biomedical Engineering, University of Houston, Houston, TX, 77204, USA; Department of Biomedical Engineering, Pennsylvania State University, University Park, PA, 16801, USA; Department of Biomedical Engineering, University of Houston, Houston, TX, 77204, USA; Department of Engineering Science and Mechanics, Pennsylvania State University, University Park, PA, 16801, USA; Department of Mechanical Engineering, University of Houston, Houston, TX, 77204, USA; Materials Science and Engineering Program, University of Houston, Houston, TX, 77204, USA; Department of Electrical and Computer Engineering, University of Houston, Houston, TX, 77204, USA; Department of Materials Science and Engineering, Materials Research Institute, Pennsylvania State University, University Park, PA, 16801, USA

## Abstract

Accurate anatomical matching for patient-specific electromyographic (EMG) mapping is crucial yet technically challenging in various medical disciplines. The fixed electrode construction of multielectrode arrays (MEAs) makes it nearly impossible to match an individual's unique muscle anatomy. This mismatch between the MEAs and target muscles leads to missing relevant muscle activity, highly redundant data, complicated electrode placement optimization, and inaccuracies in classification algorithms. Here, we present customizable and reconfigurable drawn-on-skin (DoS) MEAs as the first demonstration of high-density EMG mapping from in situ-fabricated electrodes with tunable configurations adapted to subject-specific muscle anatomy. The DoS MEAs show uniform electrical properties and can map EMG activity with high fidelity under skin deformation-induced motion, which stems from the unique and robust skin-electrode interface. They can be used to localize innervation zones (IZs), detect motor unit propagation, and capture EMG signals with consistent quality during large muscle movements. Reconfiguring the electrode arrangement of DoS MEAs to match and extend the coverage of the forearm flexors enables localization of the muscle activity and prevents missed information such as IZs. In addition, DoS MEAs customized to the specific anatomy of subjects produce highly informative data, leading to accurate finger gesture detection and prosthetic control compared with conventional technology.

Significance Statement The anatomical mismatch between the existing electromyographic (EMG) multielectrode arrays (MEAs) and target muscles leads to missing relevant muscle activity, highly redundant data, complicated electrode placement optimization, and inaccuracies in classification algorithms. Due to the fixed configuration of those MEAs, it is almost impossible to reconfigure them to match each individual's unique muscle anatomy, which is critical for physical medicine, prosthetic control, sports physiology, and rehabilitation research. This work demonstrates drawn-on-skin (DoS) MEAs as a paradigm-shifting approach to address this crucial challenge. Drawing new/erasing electrodes (without repositioning the array) allows for on-demand tunability to fully capture the spatial extent of EMG activity and improve classification. The DoS MEAs enable large-area, tunable-density, and customizable electrophysiological mapping for personalized care and treatment.

## Introduction

Accurate anatomical matching for patient-specific electromyographic (EMG) mapping is crucially needed yet challenging in various medical disciplines, such as physical/clinical medicine, prosthetic control, sports physiology, and rehabilitation (1–5). However, all the existing multielectrode arrays (MEAs), which are prefabricated using various approaches with fixed electrode configurations, are almost impossible to match each individual's unique muscle anatomy. Usually, the electrodes are repositioned in a trial-and-error manner to perform iterative measurements of muscle activity (6–15). The typically utilized conventional high-density MEAs are indiscriminate to the spatial arrangement of muscles with varying geometries and cannot be reconfigured in situ to the appropriate number and specific positions of electrodes to offer the most informative data, which is a significant challenge to overcome (14, 16, 17). In addition to highly redundant data/missed information (8, 18–20), the anatomical mismatch between the existing MEAs and target muscles also results in electrode shifts (21, 22) and motion artifacts (23, 24), further reducing the overall quality of surface EMG mapping. Devices with more deformable electrodes (25–35) could potentially be useful or repurposed for reconfiguration to some extent, but they were not designed nor are readily feasible to particularly solve the anatomical mismatch issue.

Here, we present anatomically coordinated, high-density EMG mapping with customizable and reconfigurable drawn-on-skin MEAs (DoS MEAs) as the first demonstration of simultaneous EMG mapping from many direct on-skin fabricated electrodes, adapted to the muscle anatomies of multiple subjects. Such high-density DoS MEAs are achieved for the first time, a significant departure from our initial invention of DoS electronics (36), with substantial advancements including in situ reconfigurability of the devices, anatomical matching of the devices to the targets, high-fidelity mapping of EMG signals, and uniform and low-skin electrode impedance of many DoS sensors. Most importantly, the reconfigurability and anatomical matching of DoS MEAs reduces data redundancy, thus improving classification accuracy for prosthetic control. The high-density DoS MEAs are fabricated in minutes with a biocompatible conductive ink based on an Ag/poly(3,4-ethylenedioxythiophene)-poly(styrenesulfonate) (Ag-PEDOT:PSS) composite, water/acrylic emulsion-based insulator, ballpens, and stencils. The DoS MEAs show minimal variability in their electrical characteristics compared to the current wearable bioelectronics, though the drawing process is performed by a human user's hand. Comparisons of motor unit propagation mapping, innervation zone localization, and continuous EMG measurements during large muscle movements portray the higher performance of DoS MEAs relative to conventional grids, which is important in both research and future clinical contexts. DoS MEAs reconfigured to the anatomy of the wrist flexors unveil the full extent of the target muscle activity, which the conventional grid and wearable bioelectronics cannot achieve due to their fixed construction. This broadened pool of neuromuscular information from DoS MEAs that are customized to each subject's flexors and extensors provides more distinguishable data and higher accuracy myoelectric control than existing MEA technologies. Our results suggest high-density DoS MEAs as a viable customizable and reconfigurable electrophysiological recording technology for patient-specific assessments, control, rehabilitation and/or treatments.

## Results

### High-density DoS MEA fabrication

**Fig. 1.**
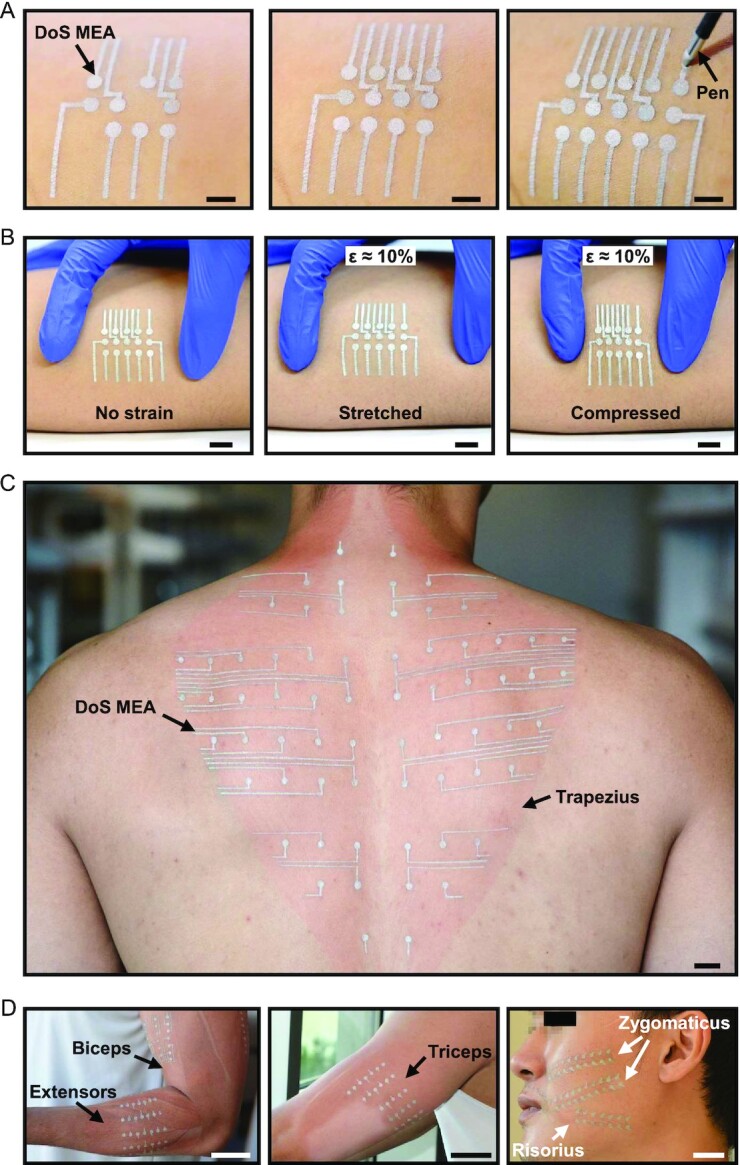
DoS electronics are configured as high-density, muscle-specific MEAs. (A) DoS MEA drawing process showing customized positions of electrodes and interconnects being fabricated (left to right) directly on the skin with a ballpen and DoS Ag-PEDOT:PSS ink (scale bar = 5 mm). (B) Deformability of DoS MEAs on skin when at 0% strain (left), 10% stretching, and 10% compressing (scale bar = 1 cm). (C) Large-area, high-density DoS MEA and interconnection pattern on the trapezius muscle of a human subject (scale bar = 1 cm). (D) DoS MEAs customized to the biceps brachii and forearm muscles (left, scale bar = 5 cm), triceps brachii (middle, scale bar = 5 cm), and facial muscles, including the zygomaticus and risorius muscles (right, scale bar = 2 cm).

The high-density DoS MEA was prepared using a highly conductive ink, insulating material, stencils, and ballpoint pens (Fig. 1A). Briefly, a stencil with the desired array configuration was prepared with a cutting machine. The Ag-PEDOT:PSS conductive ink was filled into a modified ballpoint pen (Supplementary Material*Appendix, Supplementary Materials and Methods*), which was then used to draw into the stencil on the electrode portions or draw on the skin without a stencil. It is noted that no skin preparation is needed for the ink to adhere to the skin, since it is partially hydrophilic (36). Unlike our previous report of electronics fabricated directly on skin (36), we present the first anatomically coordinated mapping of muscle activity with customizable and reconfigurable high-density DoS MEAs, which is a paradigm shift of the typical iterative process used for optimizing EMG electrode placement. The DoS MEAs can be first fabricated in any desired shape of electrode arrangements, electrode sizes, low/high-densities, with/without drawn interconnects, and then altered (by erasing and drawing in new positions) based on the drawer's intuition to capture activations specific to the target muscle. This shift from the typical approach, which is indiscriminate of the muscle anatomy, ensures that the fewest number of channels are used to reveal all the relevant muscle activations from their corresponding anatomical positions, leading to low redundancy data and, thus, improved classification of hand gestures and prosthetic control, as one example. Furthermore, this approach ensures that critical information for muscle treatments, such as innervation zones (IZs) and activation at the muscle belly, is not missed. It should be noted that when the DoS MEAs are fabricated with interconnects, they require an additional insulation material to avoid capturing signals from the interconnect lines. After drawing in the electrodes (*Supplementary Materials* Appendix, Fig. S1) with the conductive ink, a water and acrylic emulsion-based insulating material (Pros-Aide, ADM Tronics) was brushed onto the interconnect portions of the stencil and dried at room temperature. Afterward, the interconnects were drawn with the conductive ink on top of the dried insulation. Depending on whether the interconnection is prefabricated, multiple approaches could be used to collect data from the DoS MEAs as described in *SI Appendix, Supplementary Materials and Methods* and *SI Appendix*, Figs. S2 and S3. The resulting array is deformable (ε ≈ 10%) on the skin, as shown in Fig. 1B, and the ink remains functional even under 30% strain (37).

Furthermore, the MEAs can be scaled to muscles with areas on the order of hundreds of square centimeters, such as the trapezius (Fig. 1C) muscle. Previous reports of high-density EMG of the trapezius muscle show only partial coverage that misses information from either the upper, middle, or lower regions (38, 39). The electrodes and interconnects of DoS MEAs are tuned to the subject's specific muscle shape, and the interelectrode distance is determined based on the muscle geometry and intended application (40). By tuning the electrode size, density, overall arrangement, and interconnect design at the point of care, the DoS MEAs were fabricated in minutes to demonstrate their adaptability to smaller muscles like the flexors and extensors of the forearm, biceps, and triceps (Fig. 1C). The arrays were even matched to the complex arrangement and shapes of some facial muscles, including the zygomaticus and risorius muscles (8, 41). Although the DoS MEAs here are shown on a healthy subject, they could easily be adapted to the limb of an amputee patient, unlike the conventional planar and flexible grids (10). It is important to note that the dimensions of the DoS array are limited to the stencil feature size and/or pen tip diameter, depending on the usage. A minimum line width of 300 µm and line spacing of ∼200 µm were reported previously (36) but can be improved by altering the pen tip diameter and stencil feature sizes.

### High-density DoS MEA impedance characterization

**Fig. 2.**
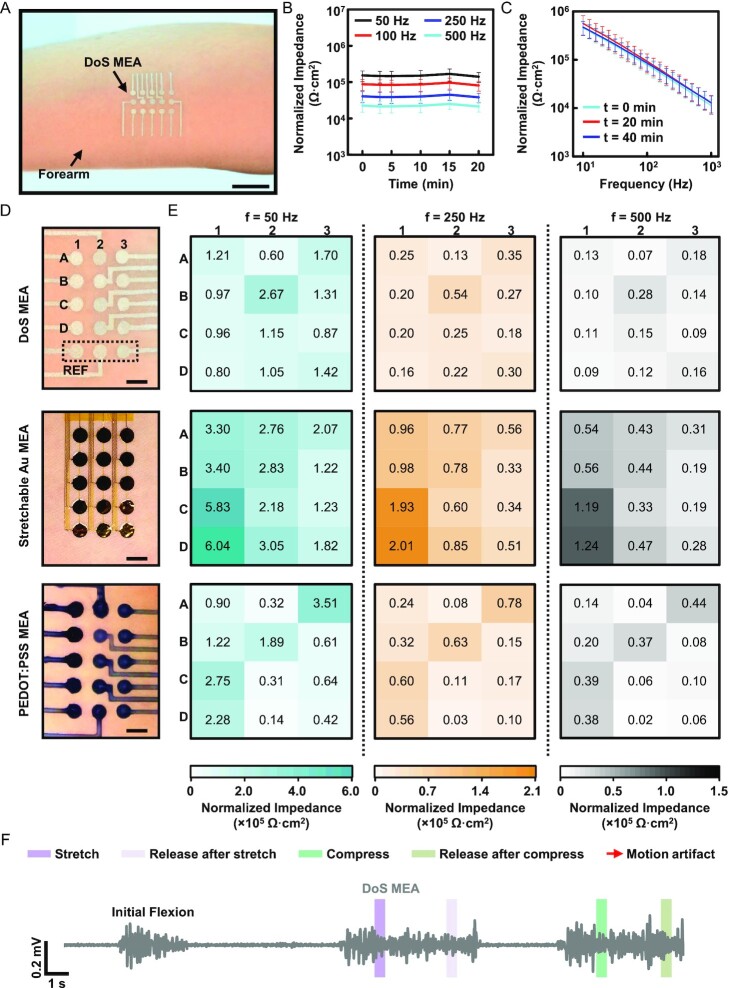
DoS MEA SEI characterization. (A) Photograph of the DoS MEA fabricated on the forearm of a subject in a 3 × 5 (row × column) array, 3 mm electrode diameter, and 5 mm interelectrode spacing (scale bar = 2 cm). (B) The average normalized SEI over time from all subjects at EMG relevant frequencies after drawing all electrodes of the DoS MEAs. Data are presented as mean ± SD. (C) Average normalized SEI spectrum from all subjects after adding additional electrodes to the DoS MEAs at 0, 20, and 40 min. Data are presented as mean ± SD. (D) Images of the DoS MEA (top), stretchable Au MEA (middle), and PEDOT: PSS (bottom) on the forearms of subjects. The labeling of “A to D” and “1 to 3” in the DoS MEA camera image indicates the rows and columns which correspond to the heatmaps (scale bar = 5 mm). (E) Average SEI heatmaps from each of the three MEAs at different measurement frequencies (50, 250, and 500 Hz). (F) EMG data recorded with three flexions of the flexor group of muscles in the forearm using the DoS MEA. The initial flexion was done without any skin deformation to the MEA. The following two flexions were performed with skin deformation, first stretching the skin around the edge of the DoS MEA and then compressing the skin.

The sensing capability of the high-density DoS MEAs was validated by measuring their impedance characteristics, and they were compared with multiple types of the existing bioelectronics (stretchable Au mesh-based MEA and intrinsically stretchable PEDOT: PSS MEA) and the conventional technology, referred to herein as the flexible printed circuit (FPC) grid (Twente Medical Systems TMSi, Enschede Netherlands). Each MEA was placed on the flexor muscle group of three subjects for skin-electrode impedance (SEI) measurements, with the DoS MEA being shown in Fig. 2A. The average SEI for all electrodes and electrode types from all subjects is shown for all impedance characterizations. In Fig. 2A, the normalized SEIs over time (after the entire DoS MEA appeared dry) at relevant physiological frequencies of EMG (50 to 500 Hz) are presented (23). Multiple impedance measurements every 5 min (until 20 min) after the MEAs were drawn indicate that the impedance remained stable, though the individual electrodes were drawn at different times. Furthermore, to mimic the scenario of adding additional electrodes to the DoS MEA and better accommodating the muscle shape and anatomy, we evaluated the impedance spectrum of individual DoS electrodes added to an MEA at 0, 20, and 40 min after the new electrodes were drawn (Fig. 2C). The difference between the impedance spectrums over time remained negligible, showing that the DoS electrodes reached a stable impedance ranging from 10^6^ to 10^4^ Ω cm^2^ (across frequencies between 10 and 1,000 Hz) within minutes of being drawn as new electrodes were added to the MEA.

The impedance spectrums of all the technologies (fabrication and dimensions in *Materials and Methods* and Supplementary Material*Appendix*, Figs. S4 to S6) were compared and revealed that the DoS electrodes show a relatively uniform normalized SEI compared to the PEDOT: PSS, Au mesh, and FPC electrodes over most of the measured range of 10 to 1000 Hz (Supplementary Material *Appendix*, Fig. S7). Average SEI heatmaps were constructed for all the MEAs (Fig. 2D and *E*) to compare the impedance of multiple devices of each (*n* = 3 per subject) technology for all subjects. The color uniformity of the heatmaps indicates the variance of the SEI across each of the MEAs. The heatmaps show minimal variation among all the electrodes in the DoS MEA, even though each electrode is drawn with a slightly different drawing speed and has a relatively more varying thickness, unlike the other electrode types. It is important to note that although this physical difference exists between the individual DoS electrodes and other electrode types, the difference is not sufficient to greatly affect the SEI. The impedance of the DoS electrodes is negligible (order of Ω) compared to the SEI (order of MΩ). In addition, it should be noted that although there are variations among the electrodes for the PEDOT: PSS and Au mesh-based MEAs, overall, they still show similar and higher SEIs, respectively, compared to those of the DoS MEA. The FPC grid (required custom D, Supplementary Material*Appendix*, Fig. S8) shows both high uniformity and lower SEI (Supplementary Material*Appendix*, Fig. S9), expected of a gel-based conventional technology. However, at the higher frequencies of EMG (>250 Hz), the electrodes of the DoS MEA show lower impedance than those of the FPC grid and other technologies. These results corroborate the use of DoS MEAs for electrophysiological signal mapping and suggest that adding electrodes to a customized DoS MEA can be done rapidly while maintaining relatively uniform impedance characteristics.

### EMG signal quality during skin deformation-induced motion

Movement artifacts are a substantial issue in EMG sensing, as noise captured from the motion can overlap with the low-frequency content comprising true muscle activity (23, 42). A further issue particularly attributed to measuring EMG signals is that certain muscles shift underneath the skin and are at different positions relative to the electrodes, depending on the level of muscle activation, and body posture (43). To evaluate the effect of relative movement between the skin and underlying muscle, a relatively stationary group of muscles (finger and wrist flexors) was chosen to ensure the muscle stayed in place while the skin was deformed. This approach (described further in the *Materials and Methods* section) ensured that minimal muscle movement relative to the skin occurred so that the artifacts could be identified as low-frequency changes to the baseline of the EMG signal during contraction, attributed solely to the skin deformation. Representative EMG signals averaged across each MEA from a single subject are shown in the Supplementary Material *Appendix*, Fig. S10. The EMG signals recorded with the DoS MEA show no artifacts during the stretching, compressing, or releasing motions (highlighted portions) while the subject flexes (Fig. 2F, zoomed-in view in the Supplementary Material*Appendix*, Fig. S10A). However, the Au and PEDOT: PSS MEAs show substantial artifacts (red arrows), as shown in the Supplementary Material*Appendix*, Fig. S10 B to E. The artifacts are clear deviations of the baseline and are relatively slower oscillations that could be removed with a high pass filter with a cutoff above 20 Hz (23).

It should be noted that although the strain distribution across the MEAs during the induced motion may not have been uniform across the array during the skin deformations, the effect of movement induced by skin deformation is clearly distributed throughout the electrodes in the Au and PEDOT: PSS MEAs to some extent, as evidenced by the averaged EMG signals. Furthermore, the same experiment was conducted using a 3 × 8 portion of the FPC grid, which showed no artifacts during any deformation (Supplementary Material*Appendix*, Fig. S10 F and G); this can be due to the strong adhesive (3). It is important to consider that the extra adhesive may only offer temporary benefits; repeated movement underneath the adhesive could cause delamination of the grid in the long term. Nevertheless, considering the longer-term wearability of DoS electronics, motion artifact-less data, and high-fidelity EMG data (described further in Supplementary Material*Appendix, Supplementary Materials and Methods* and Supplementary Material*Appendix*, Fig. S11), DoS MEAs are potentially well-suited for the dynamic, real-world ambulatory measurements.

### High-density DoS MEAs for muscle activity assessments

**Fig. 3.**
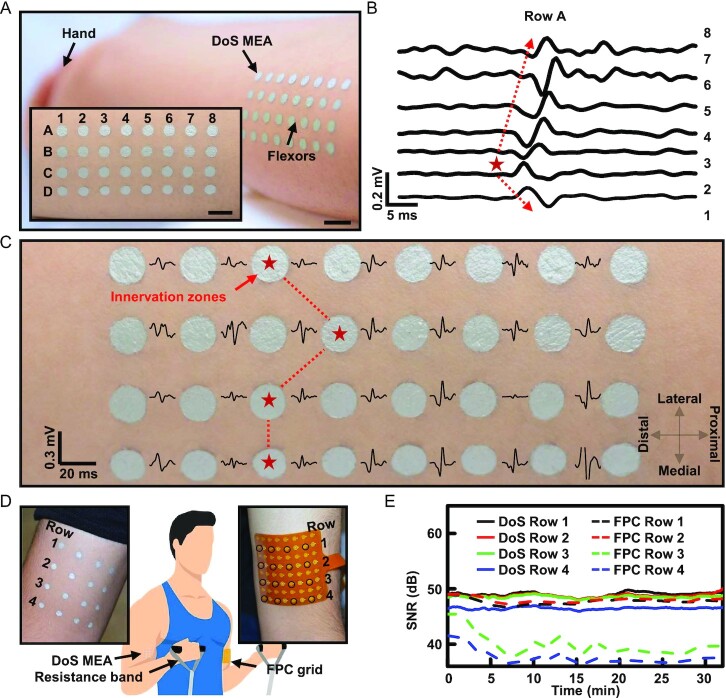
High-density DoS MEA usage for muscle activity assessments. (A) High-density DoS MEA on the flexor muscle group of a subject, inset shows the orientation of the layout (“A to D” for rows and “1 to 8” for columns, scale bar = 1 cm) for motor unit propagation mapping and innervation zone localization (scale bar = 2 cm). (B) Propagation map of a single row of the high-density DoS MEA. The change in the inflection of the wave in the third trace from the bottom (indicated by the red star) denotes the innervation zone, and the red arrows indicate the characteristic “V” pattern indicating propagation of the MUAP in different directions from the innervation zone. (C) Complete propagation maps for the entire DoS MEA and innervation zone band indicated by the red stars connected with dotted lines. (D) Setup for DoS MEA and conventional FPC grid comparison of EMG measurement during seated resistance band bicep curls. The labeling of the rows is used to calculate average EMG signals across each of the rows of the respective MEAs for signal quality examination. (E) Signal-to-noise ratios of averaged EMG signals from each row of the DoS MEA and FPC grid during 30 min of exercise.

Capturing the activity at the sites where nerve terminal branches synapse with muscle fibers, i.e., IZs, can help improve the understanding of the muscle activity and morphology in normal and pathophysiological conditions (3, 5). One application of high-density EMG is to localize the IZs of muscles as potential therapeutic targets to treat movement disorders, dystonia, and spasticity (5, 16, 44). The IZs are located through the study of motor unit action potential (MUAP) propagation. The DoS MEAs can be tuned to have varied electrode densities (low to high) and capture motor unit activity when fabricated in high-density formats (3, 45). High-density MEAs usually have ≥32 channels, ≤5 mm electrode diameter, and ≤10 mm interelectrode spacing (45). The DoS MEA, configured in a high-density format matching the dimensions of the commercial FPC electrode (Supplementary Material*Appendix*, Fig. S12), was placed on the wrist flexors (Fig. 3A) of three subjects again. Representative results are shown in Fig. 3B. The propagation results from row A (Fig. 3B) indicate a possible innervation zone among the lower channels, oriented closer to the wrist (Fig. 3C) (46). The other rows (B, C, and D) of the array show similar propagation maps (Supplementary Material*Appendix*, Fig. S13), which are in accord with row A in terms of the spatial locations of the possible IZs. The localization of all IZs from each row of the grid reveals an IZ band, representing the collective sites of motor unit innervation. From these high-density mapping results obtained with the DoS MEA, the average muscle fiber propagation velocity was calculated to be 6.33 m/s (similar to the values reported in literature (3, 40)), and individual motor units were detected by the DoS MEA as described in the Supplementary Material*Appendix, Supplementary Materials and Methods* and Supplementary Material*Appendix*, Fig. S13. As a comparison, the FPC grid was also placed in the same location (Supplementary Material*Appendix*, Fig. S14) to detect motor units. The results obtained with the high-density DoS MEA are promising for therapeutic applications in muscle recovery and prosthetics.

In another comparison of the DoS MEA (in a low-density format) and FPC grid in a manual muscle test, we evaluated the quality of the EMG signal over time during substantial movement of the biceps brachii muscle (Fig. 3D). Such large muscle movements under the skin are particularly relevant to exercise and sports physiology (45). Although the adhesive of the FPC grid is quite strong upon application, over just a few repetitions of the bicep contraction during seated dynamic bicep curls with a resistance band, a portion of the grid nearer to the crook of the elbow delaminated (indicated by the red arrow in the Supplementary Material*Appendix*, Fig. S15). An image of the setup is also provided in the Supplementary Material*Appendix*, Fig. S15. The averaged EMG signals (per row) from the lower rows (FPC grid Row 3 and 4) showed an overall lower SNR (<45 dB, calculation described in the Supplementary Material*Appendix, Supplementary Materials and Methods*) compared to those of the top rows of the grid and all the rows of the DoS MEA as plotted in Fig. 3E. In applications where large muscle movements cause substantial deformation at the skin surface, such as the above manual muscle test, the DoS MEAs appear as a viable alternative to the conventional grids.

Customizing the electrodes to the muscle anatomy can offer the appropriate resolution (1, 41, 47) and better classification accuracy from pattern recognition algorithms without creating redundancies (3, 8). Redundancies in EMG data are interference signals that decrease the differentiability of the data for classification. All of the current wearable bioelectronics and conventional technologies used for surface EMG are fixed and indiscriminate in their construction. Considering that most prosthetics are fitted based on the underlying remaining muscle activity and that those activities are detected by placing and repositioning electrodes using a trial-and-error approach (14, 16, 17), DoS electronics exclusively enables the development of reconfigurable MEAs to map all relevant spatial information at the point of care. It should be noted that electrode shifts, which occur during repositioning of the prosthetic socket relative to the electrodes, could also be avoided with DoS MEAs as they remain in position when the sockets are donned/doffed. As an example of customizing and reconfiguring DoS MEAs, we iteratively altered the arrangement of DoS electrodes relative to the commercially available FPC grid and analyzed the spatial features of each arrangement. The FPC grid used here served both as a reference to fix the position of the DoS electrodes and as an example of an indiscriminate, prefabricated device. It should be noted that the FPC grid would not be necessary in practice, and it is only used here for demonstration. Changing from 1 arrangement to another (from arrangement 1 to 3) meant that the misplaced DoS electrodes were erased (using a wet cotton swab or paper towel), and new electrodes were drawn into the positions for the next arrangement. The wires for the new positions of electrodes (depending on whether interconnection lines were drawn) could easily be attached. For example, after fabricating the new electrodes, interconnection lines could be drawn without a stencil and subsequently have wires attached on top or wires could be directly attached onto the new electrodes (as described in the*Materials and Methods* section) without needing to create an entirely new MEA.

**Fig. 4.**
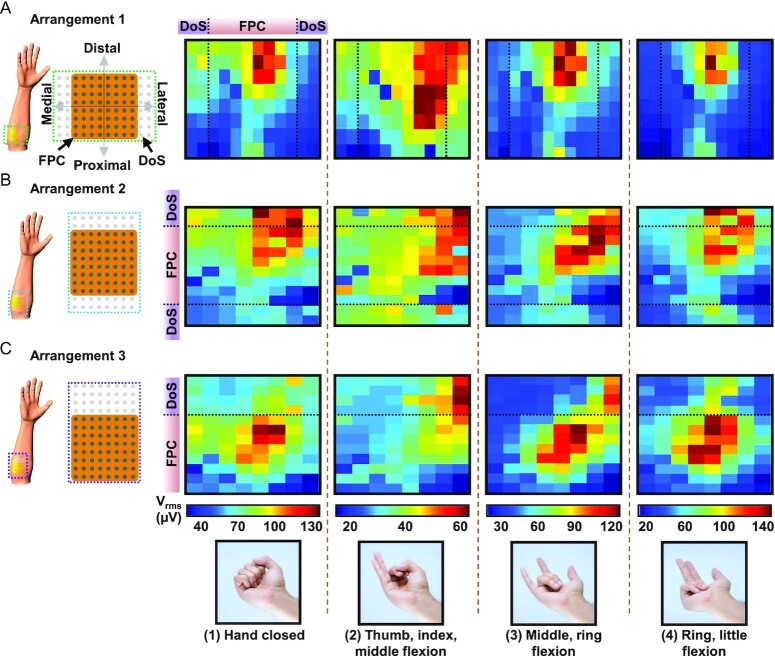
Reconfigurable DoS MEAs implemented with a conventional grid for muscle activity localization during hand flexions. (A) V_rms_ heatmaps of EMG signals acquired from DoS electrodes arranged in 8 × 2 arrays beside the FPC grid to cover the forearm in lateral and medial directions to confine the center of activity during four different hand gestures, including (1) hand closed; (2) thumb, index, middle flexion; (3) middle, ring flexion; and (4) ring, little flexion. These gestures are shown along the bottom of the whole figure. (B) V_rms_ heatmaps of EMG signals acquired from DoS electrodes arranged in 2 × 8 arrays beside the FPC grid to cover the forearm in proximal and distal directions. (C) V_rms_ heatmaps of EMG signals acquired from DoS electrodes arranged in a 4 × 8 array beside the FPC grid to cover the forearm in the distal direction.

In Fig. 4A, the FPC grid position was fixed on the wrist flexors, while the DoS MEAs were drawn in different spatial positions to determine the extent of the muscle excitation during different finger flexions. Those included (1) hands closed; (2) thumb, index, middle flexion; (3) middle, ring flexion; and (4) ring, little flexion (details in the *Materials and Methods* section) as shown along the bottom of Fig. 4. For reference, a subject's hand in a relaxed state is shown in Supplementary Material*Appendix*, Fig. S16. In Fig. 4A, the DoS electrodes were arranged in 8 × 2 arrays beside the FPC grid to improve circumferential coverage of the forearm. The DoS electrodes increase the number of channels and spatial area, supplementing the FPC grid. The heatmaps have vertical dashed lines, which indicate the edges of the activity recorded from the FPC grid. In this arrangement (arrangement 1), the EMG activities recorded from the DoS electrodes are to the left and right of the dashed lines on either side of the heatmaps in Fig. 4A. For gestures (1), (3), and (4), the voltage maps show a central pattern of activity that is more distal than proximal to the body in the upper portion of the map. The heatmap for gesture (2) shows activity that extends in the proximal direction, and the additional rows of DoS electrodes reveal activity in the lateral direction, which the FPC grid misses. The DoS electrodes consistently reveal additional regions of muscle activity for this particular gesture, even when the DoS array is reconfigured in arrangement 2 (Fig. 4B) and arrangement 3 (Fig. 4C). However, all the maps in Fig. 4A do not show discernable edges of the muscle activity, with potentially missed activity that is more distal/proximal relative to the mapped area.

In another attempt to better localize the center of muscle activity (Fig. 4B), the DoS electrodes were drawn along the length of the forearm in 2 × 8 array formats on either side of the FPC grid. The heatmaps reveal more information due to the DoS electrodes being more distal than in the previous arrangement. The horizontal dashed lines indicate the edges of the FPC grid in these maps (Fig. 4B). Above and below the dashed lines represent the activity recorded from the DoS MEAs. Still, arrangement 2 falls short in that there continues to be more activity along the length of the forearm, even more distal to the current electrode layout. The heatmaps in Fig. 4C finally constrain the activity of gestures (1) and (4), with the DoS electrodes being reconfigured to be entirely on one side (more distal) of the FPC grid. An image of the setup is shown in the Supplementary Material *Appendix*, Fig. S17. In the heatmaps in Fig. 4C, all the data above the dashed line is from the DoS MEA. Arrangement three has the best configuration of DoS electrodes compared with the other arrangements since the center of activity can be more clearly identified after tuning the electrodes to better positions. Still, it could be further improved with more rearranging of the DoS electrode positions to define the full spatial extent of the activity for gestures (2) and (3). It should be noted that although the DoS MEAs are configured in rectangular array layouts in this example, determining the full spatial extent may not require uniformly arranged layouts of electrodes and instead could require arbitrarily shaped MEAs, which cannot otherwise be achieved on demand by prefabricated grids after being placed on the skin. Through these various arrangements, the reconfigurability of DoS MEAs illustrates the ease of revealing further spatial information, which could be used to better evaluate the function of muscles in both healthy and amputee patients without greatly increasing the redundancy of the data. This approach also enables iterative localization of the center of activity in the activation maps, which could be used as highly informative image inputs to convolutional neural networks for gesture classification (48). Furthermore, it is important to acknowledge that although individual sEMG electrodes or the use of additional grids may offer some level of customizability or improved areal coverage (20), they do not simultaneously offer the benefits of DoS MEAs, including movement artifact-less recording, gel-free and imperceptible wearing, simple/inexpensive fabrication, deformability, ultra-conformal contact, and long-term usage (36), all while minimizing the redundancy in EMG data and reducing the number of channels. In addition, this demonstration highlights the potential cooperative use of DoS MEAs with existing MEAs as a step toward more personalized medical sensing.

### Customized DoS MEAs for finger gesture classification and prosthetic hand control

**Fig. 5.**
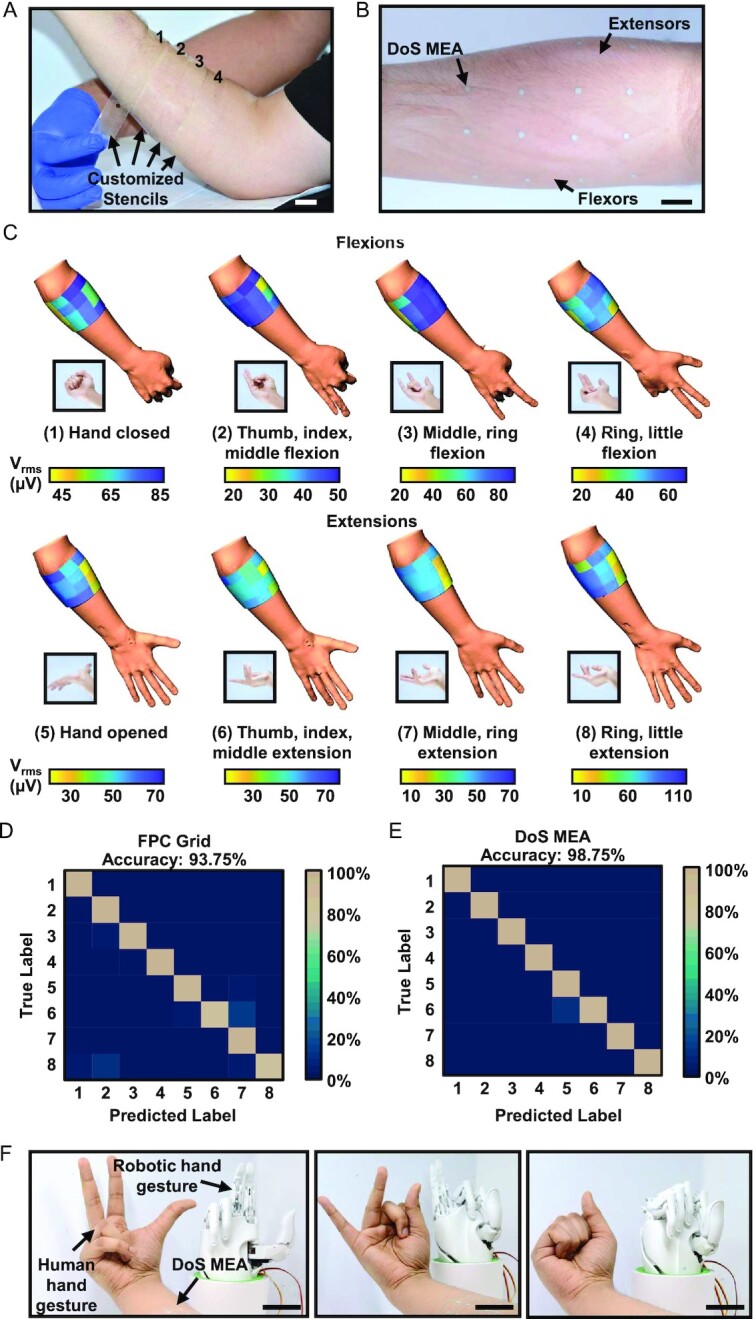
Subject-customized DoS MEAs for finger gesture classification and prosthetic hand control. (A) Custom stencils of linear eight-electrode arrays placed as four rows (to form a 4 × 8 array) around the varying circumferences (indicated by the four labeled positions) of the forearm of a subject (scale bar = 2 cm). (B) Camera image of a completed and customized DoS MEA over the flexor and extensor groups for flexion and extension-based finger gesture classification (scale bar = 2 cm). (C) V_rms_ feature maps of different gestures on lateral views of the forearm. (D) The confusion matrix from a linear discriminant analysis (LDA) classifier after offline analysis of EMG data obtained with two FPC grids having 128 channels. These grids only covered a portion of the circumference of the forearm. The numbers on the axes correspond to the labels in the feature maps above. (E) Confusion matrix from a LDA classifier after offline analysis of EMG data obtained with DoS MEAs having 32 channels. The DoS MEAs covered the entire circumference of the forearm. The numbers on the axes correspond to the labels in the feature maps above. (F) Near real-time control of a prosthetic hand by a human subject wearing the customized DoS MEA to mimic ring, little flexion (left, scale bar = 5 cm); thumb, index, middle flexion (middle, scale bar = 5 cm); and hand closed (right, scale bar = 5 cm).

Each individual's unique anatomy calls for customizable and reconfigurable sensing platforms for accurate, personalized care. Various studies demonstrate the importance of EMG arrays customized to the anatomy of the target muscles with varying electrode dimensions, spacing, and overall sizes (7–9, 41, 49). The customizability and reconfigurability of the DoS MEAs reduce data redundancy and improve classification accuracy for prosthetic control, distinguishing this work from the existing studies, all of which do not demonstrate reconfigurability. The completed DoS MEAs made with customized stencils (Fig. 5A and Supplementary Material*Appendix*, Fig. S18) covered both the wrist/finger flexors and extensors, as shown in Fig. 5B. Each subject was asked to perform the four aforementioned gestures along with the following extensions, including (5) hand open; (6) thumb, index, middle extension; (7) middle, ring extension; and (8) ring, little extension. Lateral views of the forearm show that across all flexions (gestures 1 to 4), the more proximal and posterior portion of the forearm shows relatively higher excitation (Fig. 5C). In addition, gestures (1), (2), and (4) show some excitation over the extensors, which is in agreement with reported literature (50, 51). For all the extension gestures, the lateral views of the forearm show consistent excitation across the group of extensors (bottom half of Fig. 5C). The medial views of the forearm for both flexion and extension gestures are shown in the Supplementary Material*Appendix*, Fig. S19. Compared to the excitation maps of the lateral view, those of the medial view show comparably less activity for both sets of gestures.

Controlling prosthetic hands with surface EMG is a promising strategy to improve the quality of life for patients with impaired mobility of limbs (6, 16). We compared using the custom DoS MEAs with two FPC grids placed next to each other (Supplementary Material*Appendix*, Fig. S20) for controlling a prosthetic hand based on the different finger gestures. Here, the V_rms_ features extracted from the EMG data were fed into a LDA classifier, both of which are among the simplest features and pattern recognition algorithms to utilize, respectively (52, 53). Details of the classification are in *Materials and Methods*. Offline analysis of the EMG data (Fig. 5D and *E*) showed higher classification accuracy obtained with DoS MEAs (98.75%) as compared to the FPC grids (93.75%). It should also be noted that this was achieved using only 32 DoS electrodes compared to the 128 FPC electrodes. This difference is likely due to the lower redundancy of the data and, therefore, higher V_rms_ feature separability, as indicated by the principal component analysis results shown in the Supplementary Material*Appendix*, Fig. S21.

Additionally, due to their placement, the FPC grids could not obtain the same spatial information as the DoS MEA, and additional grids would be necessary, further complicating acquisition and postprocessing. With an online classifier and the customized DoS MEA, the subjects were able to control a prosthetic hand in near real-time, as shown in Fig. 5F. Taken together, the surface EMG and classification results from the customized DoS MEAs are promising for use in both healthy and patients with disabilities for accurate prosthetic control.

## Discussion

The DoS MEAs presented in this work are the first demonstration of high-density electrophysiological signal mapping with devices fabricated in situ. The approaches for customizing the DoS MEAs, collecting data from them, and reconfiguring them to obtain the highly informative EMG data indicate a feasible practice that could be performed by anyone that has a general understanding of human muscle anatomy. In addition, future computer-aided simulation and design of the geometries of the DoS MEAs could provide improved performance. On top of overcoming the limitations of high redundancy in EMG data and fixed construction of the existing MEAs, DoS MEAs bring several advantages, including relatively uniform impedance characteristics despite the manual drawing process, motion artifact-less EMG data in the presence of skin-deformation-induced motion, and detection of critical neuromuscular properties in both high- and low-density formats with high-fidelity EMG signals. Importantly, the ability to customize the DoS MEAs and reconfigure them is a method that most naturally suits the iterative manner by which the optimal positions of EMG electrodes are typically determined (1). Although the drawing process is completed with a stencil, purely hand drawing without a stencil is also possible, particularly when the device geometry is not critical to its performances. Other drawing methods, such as contoured 3D printing, could also be feasible. DoS MEAs, as a paradigm-shifting technology, could be implemented as a large-area, tunable-density, and in situ reconfigurable electrophysiological mapping technology for personalized medicine in muscle treatments, myoelectric control, sports physiology, and human-machine interfaces.

## Materials and methods

### Materials

Ag flakes (10 µm size, 99.9% trace metals basis, 327,077), and poly(ethylene glycol)-block-poly(propylene glycol)-block-poly(ethylene glycol) (Pluronic P-123,435,465) were purchased from Sigma–Aldrich and used without further modification. PEDOT: PSS (PH 1000) was from Ossila Limited. The insulation material (Pros-Aide) was a water-based acrylic emulsion from ADM Tronics. The conductive wire glue (made from graphite, polyvinyl acetate, and water) was from Anders Products.

### Conductive ink preparation

The DoS conductive ink was prepared by first making the highly conductive PEDOT: PSS solution and then adding in the Ag flakes. First, the PEDOT: PSS solution was prepared by stirring 10 wt.% P-123 into the commercial PEDOT: PSS solution for 12 h at room temperature (∼ 22°C) at 800 rpm. Afterward, the prepared solution was stored at ∼4°C in a refrigerator. Prior to adding Ag flakes, the PEDOT: PSS solution was taken out of the refrigerator and stirred for 1 to 2 min. Then the corresponding amount of Ag flakes (1:2 weight ratio, Ag flakes: PEDOT: PSS solution) in the form of powder was added to the vial, and the PEDOT: PSS solution was added to the vial, and the mixture was stirred on a magnetic stirrer for about 1 h. The resulting ink was ready to use after the stirring, but it could be stirred more if any visible Ag flakes powder remained.

### DoS MEA fabrication on skin with custom interconnection schemes

For interconnection schemes that were drawn (with or without a stencil) or when wires from a data acquisition (DAQ) system were directly attached to DoS electrodes, the approach described here was utilized. The DoS MEAs were prepared using modified ballpoint pens, stencils, the conductive ink, Pros-Aide, stainless steel wires (790,900, A-M systems), conductive wire glue, electrode collar adhesive (TD23, Refa), and tape (Magic Tape, 3M). The fabrication of the stencils is described in the Supplementary Material *Appendix*,*Supplementary Materials and Methods*. The skin of the subject was wiped with an alcohol prep pad for a few seconds, and the stencil was applied. If the stencil did not have interconnections, the electrodes were drawn into the circular parts of the stencil (Supplementary Material *Appendix*, Fig. S1). A stainless steel wire was laid on top of the electrode, and the electrode collar adhesive was laminated on top to connect the electrodes directly to the DAQ system. Finally, a drop of DoS ink was placed inside the hole of the electrode collar adhesive to sandwich the wire (Supplementary Material *Appendix*, Fig. S2). If the stencil had interconnections, the first step was to draw the electrodes over the circular parts of the stencil (Supplementary Material *Appendix*, Fig. S1) and leave them to dry for 3 to 5 min. Next, the insulation material (Pros-Aide) was brushed onto the interconnect lines exposed in the stencil. After the insulation material became clear and slightly tacky (5 to10 min), the interconnection lines were drawn over the Pros-Aide and left to dry another 3 to 5 min. The stencil was removed slightly before all the DoS ink appeared dry. A stainless steel wire was taped to the skin, with the exposed part laying over the end of the DoS interconnect line to wire the electrodes to the DAQ. Conductive wire glue was painted onto two portions of the exposed wire over the interconnect (Supplementary Material*Appendix*, Fig. S2) to clamp the wire down to the skin. A hairdryer (Conair) was held at a low setting for 1 min to cure the glue. Then one more layer was drawn with the DoS ink on top of the wire glue to sandwich the wire.

### DoS MEA fabrication on skin with prefabricated interconnection schemes

The following approach was utilized if the interconnection scheme was prefabricated (i.e., in contexts when the design can be ascertained before the in situ application). The DoS MEAs were prepared using modified ballpoint pens, stencils, the conductive ink, Pros-Aide, and the prefabricated interconnection film. The fabrication of the stencils is described in the Supplementary Material*Appendix, Supplementary Materials and Methods*, and the fabrication of the interconnection scheme is described later in this section. The skin of the subject was wiped with an alcohol prep pad for a few seconds, and the stencil was applied. The electrodes were drawn over the circular parts of the stencil (Supplementary Material*Appendix*, Fig. S1) and left to dry for 3 to 5 min. Next, the insulation material (Pros-Aide) was brushed onto the interconnect lines exposed in the stencil. After the insulation material became clear and slightly tacky (5 to 10 min), the interconnection lines were drawn over the Pros-Aide and left to dry another 3 to 5 min. The stencil was removed slightly before all the DoS ink appeared dry. Prior to applying the prefabricated interconnection film to the skin, the exposed PI film and unused interconnection lines were covered with Pros-Aide. After 5 to 10 min (the film appeared clear and was slightly tacky), the interconnection film was laminated to the skin with the interconnection film aligned to the DoS interconnection lines. The fabrication of the interconnection film is described in the following. A glass slide was cleaned using acetone, isopropyl alcohol (IPA), and DI water. A ∼2 μm thick polyimide (PI-2545, HD Microsystems) film was spin coated on the glass slide. Then 5 nm/100 nm thick Cr/Au layers were deposited via an e-beam evaporator. The metal layers were then patterned by photolithography and wet etching. The film was released from the glass slide using buffered oxide etchant (BOE, 6:1, Transene Company Inc.). After releasing, the metal interconnect was connected to a custom-made PCB through an ACF cable for data measurement. The PCB had male headers that could be attached to a DAQ system with breadboard wires.

### SEI characterization

All the procedures were approved by the Institutional Review Board of the University of Houston, TX (USA) and informed consent was obtained (Protocol 2765). To validate the sensing capabilities of the DoS MEAs, they were compared with multiple types of the existing bioelectronics and the conventional technology. Specifically, we first compared the impedance characteristics of the DoS electrodes with those of a structurally engineered stretchable Au mesh-based MEA, a 3D printed and intrinsically stretchable PEDOT: PSS MEA, and a flexible printed TMSi grid. The fabrication processes of the stretchable Au mesh and printed PEDOT: PSS-based MEAs are depicted in the Supplementary Material*Appendix*, Figs. S4 and S5, respectively. The dimensions of the arrays are labeled in Supplementary Material*Appendix*, Fig. S6. It should be noted that the number of electrodes, diameters, and interelectrode distance was consistent among the wearable MEAs, apart from those of the FPC grid. Specifically, the DoS, PEDOT: PSS, and Au MEAs each had electrodes that were 3 mm in diameter and spaced 5 mm apart, all arranged in a 3 × 5 (row × column) grid. The interelectrode spacing is reported center-to-center throughout the rest of this work. A custom connection scheme was developed to ensure that all devices were evaluated in a similar manner (Supplementary Material*Appendix*, Fig. S8) to acquire data specifically from the FPC grid. The only additional connection needed for the SEI measurements from the FPC grid was a proprietary TMSi cable that connected to the contact pads of the conventional grid and an adapter that was fitted with breadboard wires. It should be noted that the FPC grid had electrodes that were 4.5 mm in diameter and had an interelectrode distance of 8.75 mm. An SEI heatmap of a 3 × 5 portion of the 64-channel FPC grid is shown in the Supplementary Material*Appendix*, Fig. S9. For all SEI measurements, the same measurement settings were used. The SEI was measured using an impedance analyzer (Multi/Autolab M204, Metrohm) that measured the impedance from 1000 to 1 Hz. A two-electrode configuration was used. The working electrode and reference electrode were connected to two electrodes in the array placed on the skin. In each row (for each type of MEA), one electrode was fixed as the reference electrode, and the working electrode was moved to another electrode (of the MEA) sequentially to create the SEI heatmaps.

### EMG DAQ

The areas of skin on which the electrodes were placed were prepared with an alcohol prep pad that was scrubbed on the skin for a few seconds. It is noted that this preparation step is not always necessary to successfully capture EMG signals. The snap electrical leads were connected to an interface board (Recording Controller, Intan Technologies) via an amplifier board (RHD2132, Intan Technologies) with unipolar input channels. In the DAQ program, a sampling rate of 2000 Hz was utilized (54, 55), and the notch filter (60 Hz) setting was turned on. A wet cuff electrode was placed on the bony portion of the wrist to serve as the ground electrode for all measurements. The bandwidth was set to 0.1 to 1000 Hz. Signals were processed with a third-order Butterworth bandpass filter, with the cutoff frequencies being 20 and 500 Hz.

### Skin deformation-induced motion during EMG sensing

The quality of the EMG signals from each MEA was determined without and with skin deformation-induced motion for the three subjects. The subjects were asked to squeeze their right hand into a fist at regular intervals, three times per trial (*n* = 10, per MEA type). After an initial flexion, their skin was manually deformed by the experimenter (at a speed of ∼2 mm/s) at opposite ends of the MEAs during the following two flexions as an extreme case of skin deformation during muscle contraction. In the second flexion, the skin around the MEA was stretched (stretching motion duration is indicated by the dark purple bar) and released (indicated by the light purple bar). In the third contraction, the skin around the MEA was compressed (compressing motion duration is indicated by the green bar) and released (indicated by the light green bar). Signals were processed with a third-order Butterworth bandpass filter, with the cutoff frequencies being 1 and 500 Hz. The lower cutoff is used here to demonstrate the effect of the induced motion.

### Innervation zone localization and MUAP detection

Electrodes were drawn into a 4 × 8 grid, each being 4.5 mm in diameter and spaced 8.75 mm apart to match the dimensions of the commercial FPC grid (Supplementary Material*Appendix*, Fig. S12) for further comparisons. For each row of the grid, a propagation map was created, and IZs could easily be identified through the changing direction of the deflection from the baseline (5). Bipolar EMG signals were first derived from the monopolar signals by taking the difference between neighboring channels along the muscle fiber direction. The most prominent MUAPs present in the surface interferential pattern were separated via blind source separation using the Joint Approximation Diagonalization of Eigenmatrices (JADE) algorithm (56). Separated components demonstrating motor unit spiking activity were selected, and spike times were determined via threshold detection to isolate spikes generated from a single motor unit. Time series EMG data were then spike-triggered averaged with respect to the determined spike-times to give the spatiotemporal representation of the MUAP, as shown in Fig. 3B. To achieve decomposition of lower amplitude MUAPs, the K-Means Clustering and Convolution Kernel Compensation (KMCKC) algorithm was utilized to determine spike times from a more diverse pool of motor units (57). Similarly, the time-series EMG data were spike-trigger averaged for each decomposed MUAP, and represented spatially, as shown in Fig. 3C. The IZs were determined by inspecting the channel where propagating MUAPs demonstrate a phase reversal.

### EMG measurement during seated resistance band curls

Each of the three subjects was asked to perform seated resistance band bicep curls while wearing a 4 × 4 DoS MEA and FPC grid (Fig. 3D) for ∼30 min with rests in between. A metronome was set to 50 bpm for 1 min sets, with  1 min breaks between sets until each fifth set. About 25 contraction/relaxation cycles were performed in each set. In the fifth and tenth sets, the subjects were given 2 min breaks. Fewer rows and channels (again in a 4 × 4 format) were used from the 64-channel FPC grid with a 17.5 mm spacing between electrodes for both MEAs to simplify the data processing. Anatomically, the long and short heads of the biceps brachii were covered by both MEAs, and the centers of the muscles slid back and forth under different regions of the MEAs. The SNR calculation is described in the Supplementary Material*Appendix, Supplementary Materials and Methods*. Only the contraction regions of the EMG data were used for processing. The plotted data shows the averaged SNR per row for each contraction over time. Signals were processed with a third-order Butterworth bandpass filter, with cutoff frequencies at 20 and 500 Hz.

### Reconfigurable DoS MEA and conventional grid setup

For all the EMG measurements performed during hand gesture experiments throughout this work, the subjects rested their arm on a table with their hand hanging slightly off but kept their hands and wrists in a neutral position to minimize any pronation/supination based artifacts (6, 43, 58). The FPC grid was placed on the belly of the flexor muscles in the forearm of three subjects, and representative results are shown. The DoS MEAs were drawn in arrangement 1 (Fig. 4A), and the data was obtained simultaneously with the FPC grid and DoS MEAs. It is assumed that the individual applying the DoS electrodes to the patient has a general understanding of the muscle anatomy and can simply fine-tune the DoS MEA by adding and removing electrodes. After analyzing the data and noting that the extent of the muscle activity seemed to extend beyond the analyzed anatomical location, another arrangement was tested. The unnecessary electrodes of the DoS MEA were wiped off with a wet cotton swab/paper towel, and DoS electrodes were drawn onto the new locations for arrangement 2. Again, after analyzing the data for arrangement 2, it seemed that activity extended in the distal direction. Arrangement 3 was made to accommodate this, in the same manner performed for arrangement 2.

### Customized DoS MEA fabrication for finger gesture classification

Stencils were designed for custom DoS MEAs that covered the entire circumference of the forearm at four positions (Fig. 5A and Supplementary Material*Appendix*, Fig. S18). Each stencil was a linear array (containing eight electrodes) with different lengths and varied spacing based on the circumference at the four positions, which were evenly distributed over the bellies of the forearm muscle groups.

### Finger gesture classification and principal component analysis

For each subject, four sessions per gesture were performed, with each session having 10 trials. Representative excitation feature maps are shown in Fig. 5C, specifically using the root-mean-square voltage (V_rms_) as the feature. The V_rms_ feature was extracted from the steady-state portion of four unique finger flexion gestures performed by the participants from both the DoS MEAs and commercial FPC grids. V_rms_ features from all finger gestures were standardized to zero mean and unit variance (Z-score) prior to principal component analysis (PCA) for visualization of cluster separation in reduced dimensions. V_rms_ features were then classified via a LDAs model. The LDA model performance was evaluated using 5-fold validation to derive the cross-validated classification loss.

### Prosthetic hand control

After offline analysis and classification were performed, the same LDA classifier was used to perform online predictions based on a model trained with the obtained data from the subjects. The output of the classification was sent to a custom Arduino script, which was written to control the prosthetic hand. EMG data was obtained in near real-time from the DoS MEAs customized to each subject who performed the gestures as shown in Fig. 5.

## Funding

C.Y. would like to acknowledge the funding support of the Office of Naval Research grants (N–-1–2480), the National Institute of Health grant (R21EB026175), and the National Science Foundation grant (CBET-1936151). F.E. would like to acknowledge the National Science Foundation Graduate Research Fellowship Program (DGE1255832).

## Authors' Contribution

F.E., M.H., S.P., Z.R., N.D., and C.Y. conceived and designed the devices and experiments. F.E., M.H., S.P., L.C., B.K., and Y.L. prepared the materials and/or fabricated the devices. F.E., S.P., L.C., and B.K. performed the characterization experiments. F.E., M.H., S.P., L.C., and B.K. performed the electrophysiological experiments. F.E., M.H., S.P., L.C., Y.L., and N.D. processed and analyzed the experimental data. F.E., M.H., S.P., and A.H.G. developed the robotic hand control experiment and application. F.E., M.H., and C.Y. wrote the manuscript. All authors reviewed the manuscript.

## Supplementary Material

pgac291_Supplemental_File
