# Supplementary material for: Customizable, reconfigurable, and anatomically coordinated large-area, high-density electromyography from drawn-on-skin electrode arrays
Source: PNAS Nexus. 2023 Jan 11;2(1):pgac291. doi: 10.1093/pnasnexus/pgac291 (PMC9837666; doi:10.1093/pnasnexus/pgac291)
Supplement: pgac291_Supplemental_File [file pgac291_supplemental_file.docx]

**
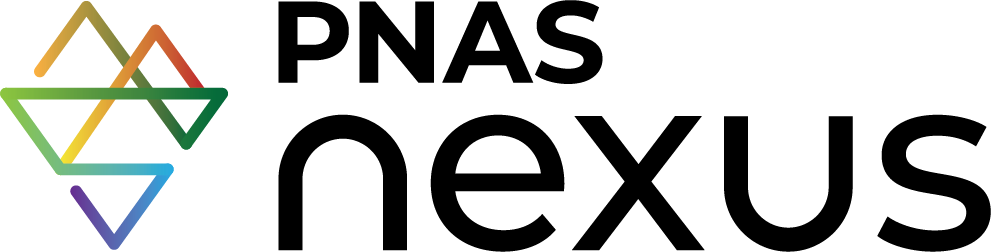
**

**Supplementary Information for**

Customizable, reconfigurable and anatomically coordinated large-area, high-density electromyography from drawn-on-skin electrode arrays

Faheem Ershad, Michael Houston, Shubham Patel, Luis Contreras, Bikram Koirala, Yuntao Lu, Zhoulyu Rao, Yang Liu, Nicholas Dias, Arturo Haces-Garcia, Weihang Zhu Yingchun Zhang, Cunjiang Yu*

*Corresponding author. Email: [cmy5358@psu.edu](mailto:cmy5358@psu.edu)

**This PDF file includes:**

Supplementary Materials and Methods

Figures S1 to S21

SI References

**Supplementary Materials and Methods**

# Ballpoint Pen Preparation.

Ballpoint pens (557154012, PEN + GEAR) were fully disassembled. The balls from the pen tips and the original inks were removed. The tips and ink barrels were thoroughly cleaned in acetone, sonicated in deionized (DI) water, and air dried. Then, the ink was injected into the emptied ink barrels via a syringe and 26-gauge needle.

# Stencil Fabrication.

The stencils were designed in AutoCAD. A cutting board was layered with one layer of packing tape (Duck). The cutting machine (Silhouette Cameo) was programmed to cut the stencils based on the designs. The stencils were removed from the cutting board and then placed onto a sticker sheet for later use.

# Fabrication of Stretchable Au MEA.

First, a glass slide was cleaned using acetone, isopropyl alcohol (IPA), and DI water. A 200-250 nm thick polyimide (PI-2545, HD Microsystems) film was made by spin coating. Then 5 nm/100 nm thick Cr/Au layers were deposited via an e-beam evaporator. The metal layers were then patterned by photolithography and wet etching. The PI was patterned by reactive ion etching (RIE, Oxford Plasma Lab 80 Plus). Finally, a layer of poly(methyl methacrylate) (PMMA) was spin coated onto the metal side to aid transfer and temporarily maintain the structure of the electrode. The electrode was released from the glass using buffered oxide etchant (BOE, 6:1, Transene Company Inc.) and then picked up using wax paper. The PMMA was dissolved using acetone. The electrode was then transferred from the wax paper to the skin.

# DoS MEA Interconnection Setup for Data Acquisition.

Unlike the typical bioelectronics, the DoS sensors and devices present the unique opportunity to make interconnection systems directly on the body using just the DoS inks. For the purposes of this work, we demonstrate wired approaches to illustrate the potential use of DoS electrode arrays in a simple manner. Using an electrode collar adhesive can allow the user to ascertain that the stainless-steel wires directly contact the DoS electrodes (*SI Appendix*, Fig. S2 *A*). The electrode collar is donut-shaped and the hole in the center allows the experimenter to confirm the wires are secured to the DoS electrodes. The cross-sectional view of the interface is shown at the bottom of *SI Appendix*, Fig. S2 *A*. This approach could be adapted to DoS interconnects, if the spacing between them is relatively large (>10 mm). Furthermore, the use of conductive adhesives can also facilitate contact between external wires and the DoS interconnects in MEAs as shown in *SI Appendix*, Fig. S2 *B*. Here we chose to use conductive wire glue as it is water-based, safe for use on the skin, and can be dried quickly. In this alternative approach, after the interconnect was drawn, the conductive wire was secured at two ends with a conductive glue (Wire Glue, Anders Products). The wire was then covered with additional DoS conductive ink across the entire length exposed to the interconnect. The cross-section of this arrangement is shown in the bottom *SI Appendix*, Fig. S2 *B* as well. For use cases in which the array is customized to the individual or the interconnection design is uncertain, the adhesive and external wire approaches are suitable. If the design of the array interconnection is known beforehand however, interconnection films could be custom manufactured through traditional microfabrication as well. An example of this is shown in *SI Appendix*, Fig. S3. The interconnection film was designed in such a pattern that it could be adapted to either side of the DoS MEA. It is noted that although extra interconnection lines were fabricated on the film, their corresponding contact pads did not have any connection to the data acquisition (DAQ) system. This approach could be used to rapidly collect data from several electrodes (tens of channels) simultaneously as the interconnecting film could be prepared with an anisotropic conductive film (ACF) cable bonded to a printed circuit board (PCB). The PCB could be connected to any data DAQ with an adapter. Depending on whether the interconnect design is predetermined, any of the aforementioned approaches would suffice for collecting data from several channels of DoS electrodes.

# Fast Fourier Transform Characteristics of EMG Data from MEAs.

The fast Fourier Transforms (FFT) of the averaged EMG signals (initial contraction) from each array are shown in *SI Appendix*, Fig. S11. The DoS and PEDOT:PSS MEAs show a similar magnitude through the lower half of the analyzed spectrum (1-500 Hz), while the PEDOT:PSS MEA shows a slightly higher magnitude in frequencies above 150 Hz. This is likely due to the higher concentration of PEDOT:PSS as the electrode material and better ionic conductivity compared to the high concentration of Ag flakes used in the DoS ink (1). The Au MEA also shows a similar frequency profile to that of the DoS MEA, except that the magnitude remains relatively lower below 150 Hz. The frequency profile of the FPC grid shows relatively higher power across the entire spectrum compared to the other MEAs, which is likely due to the application of a conductive gel to the surface of the grid prior to placement on the target muscle. For prosthetic control, wearable grids may be worn for hours, to days, weeks, or months, making gels a generally undesirable additive since they can dry out quickly (2).

# Muscle Fiber Propagation Speed and Detection.

To calculate the muscle fiber conduction velocity, first, the differences in timing of the sequential positive (or negative) peaks across the columns in each row (A, B, C, D) from the propagation maps (*SI Appendix*, Fig. S13) were determined. The distance between each electrode was divided by each of the differences and the values were averaged and converted to m/s. The number of motor units captured using the DoS MEA and FPC grid (placed on the same muscle, *SI Appendix*, Fig. S14) were compared. The number of motor units for a representative subject detected using the DoS MEA and FPC grid were 3 and 17, respectively. This difference in the number of motor units detected could be due to the highly optimized and locally shielded data acquisition interface used with the FPC grid as compared to the current unshielded data acquisition approach with DoS electronics. Another important consideration for MUAP detection is that by knowingly targeting the belly of the muscle, the number of electrodes required could be reduced, which reduces the computation performed in postprocessing (3).

# SNR calculation for EMG signals.

To calculate the SNR for each of the sensor types, first the power spectral density estimate was obtained using Welch’s method in MATLAB. The parameters for the pwelch function were chosen to be a 400-point Hanning window and a 50% overlap. Signals in the 20-500 Hz range of the power spectrum represent the “signal” in the SNR calculation and the power was summed over those frequencies and normalized to be in units of dB. The noise was averaged from the rest of the power spectrum (500-1000 Hz) and represent the “noise” in the SNR calculation. The following formula was used to convert the ratio of the signal and noise to power in dB:

$SNR= 10*\frac{P_{(s)}}{P_{(n)}}$ (S1)

where P_(s)_ is the power of the signal and P_(n)_ is the power of the noise.

**Supplementary Figures**

**
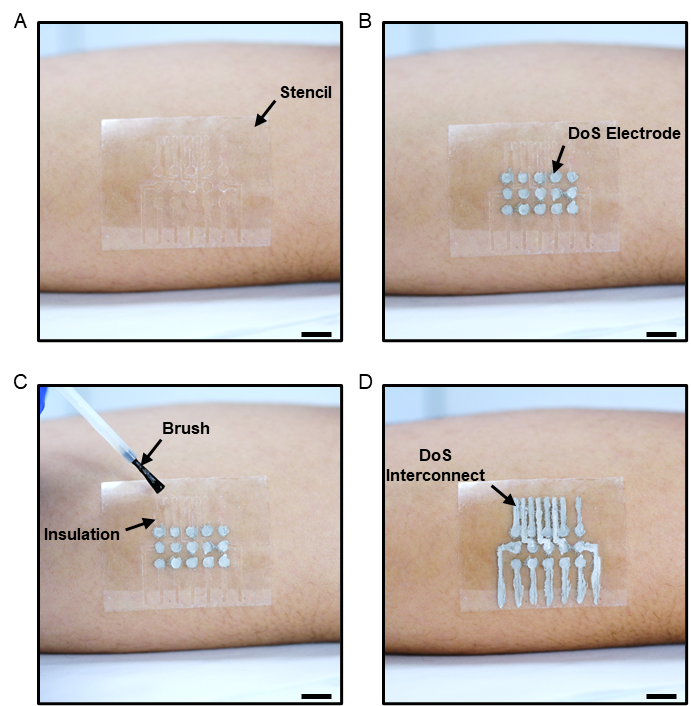
**

**Fig. S1.** DoS MEA fabrication. (*A*) Tape based stencil laminated onto the forearm of the human subject. (*B*) DoS conductive ink drawn into the positions for the electrodes in the MEA. (*C*) Brushing of the acrylic-based insulating material (Pros-Aide, ADM Tronics) onto the interconnect regions of the MEA. (*D*) After a few minutes of drying, the DoS conductive ink was drawn on top of the insulating material and left to dry for a few more minutes. Scale bars = 1 cm.


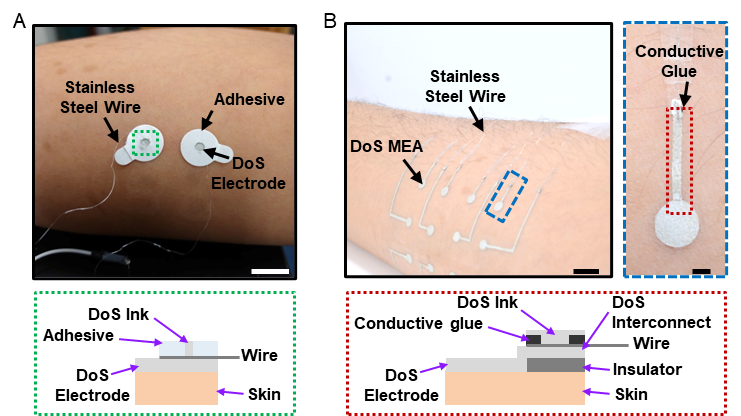


**Fig. S2.** Data acquisition approaches from customized DoS electrodes and MEAs. (*A*) Electrode collar adhesive used to secure the stainless steel wires from the data acquisition system directly to the DoS electrode (scale bar = 1 cm). Below is a schematic of the cross section. (*B*) Conductive wire glue (Wire Glue, American Science and Surplus) used to fix the stainless-steel wire on the interconnects of DoS MEAs (scale bar = 2 cm). Zoom in shown on the image on the right (scale bar = 2 mm). The glue was placed in two positions to clamp the wire down and then DoS conductive ink was used to cover the entire exposed portion of the wire and dried wire glue.

**
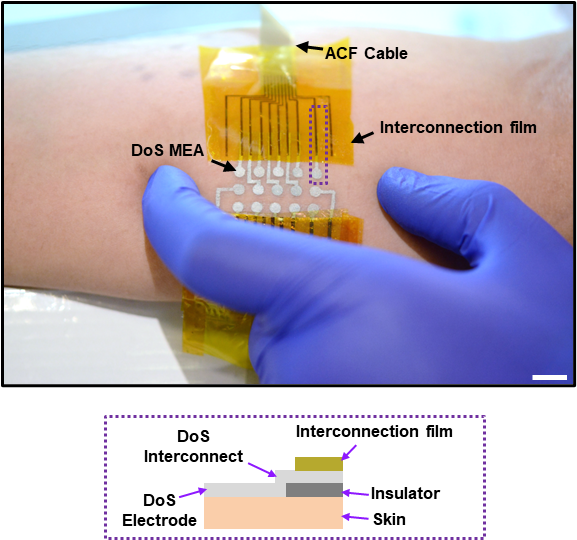
**

**Fig. S3.** Data acquisition approach from DoS MEAs with prefabricated interconnections. The interconnection films were laminated onto the interconnects of the DoS MEAs. Since the DoS MEA did not have a symmetrical arrangement in this instance, the interconnection film was fabricated so that it could be laminated to either side of the DoS MEA. Scale bar = 1 cm.

**
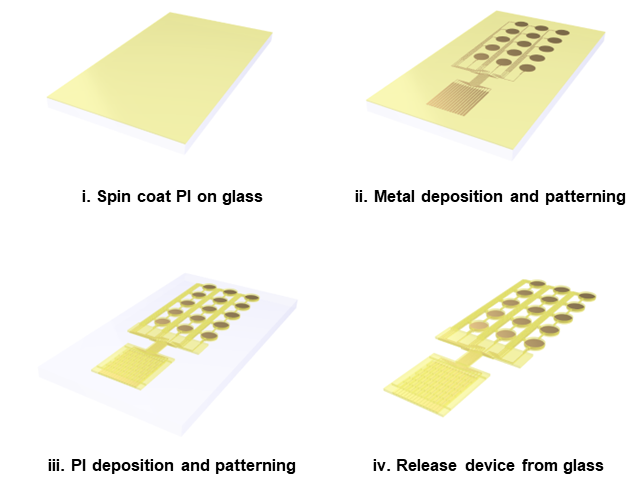
**

**Fig. S4.** Fabrication process for the stretchable Au MEA.

**
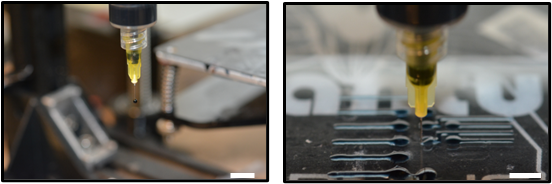
**

**Fig. S5.** Fabrication process for the printed PEDOT:PSS MEAs. The image on the left (scale bar = 1 cm) shows the PEDOT:PSS droplet forming at the tip of the needle attached to a syringe of a custom-built pneumatic extrusion printer. The image on the right (scale bar = 1 cm) shows the printing process.

**
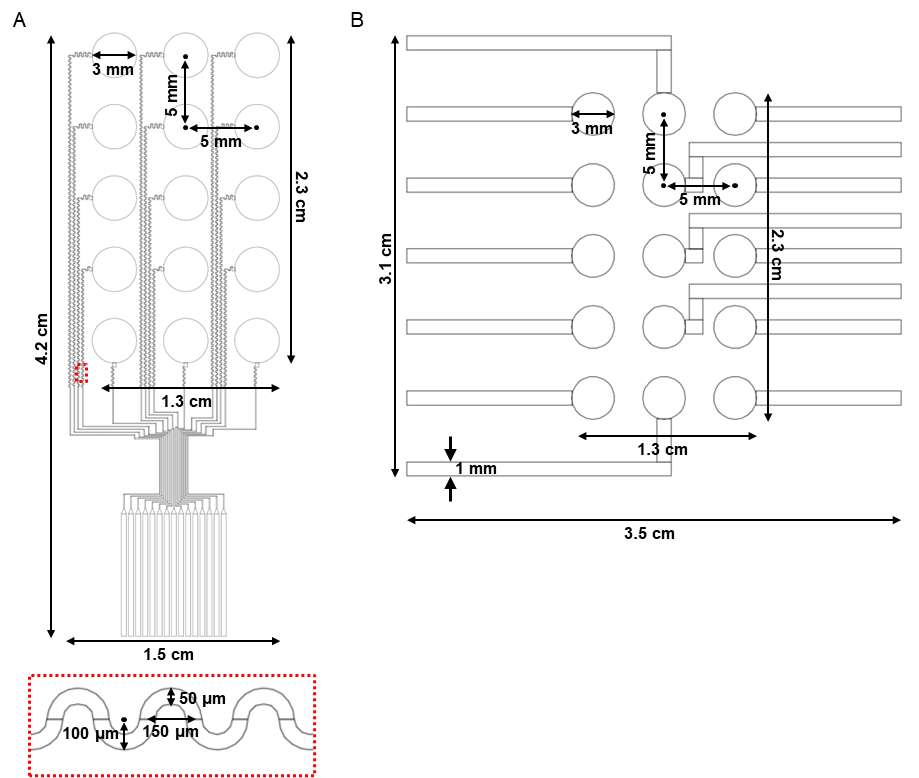
**

**Fig. S6.** Detailed geometrical dimensions of the MEAs. (*A*) Dimensions of the stretchable Au MEA. Inset shows dimensions of the serpentine pattern for the interconnects. (*B*) Dimensions of the DoS and PEDOT:PSS MEAs.

**
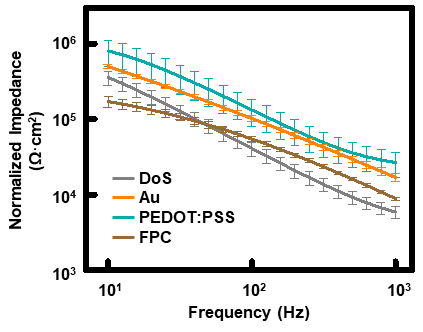
**

**Fig. S7.** Comparison of the normalized skin-electrode impedance between the DoS, Au, PEDOT:PSS MEAs, and FPC grid. Data are presented as mean ± s.d.

**
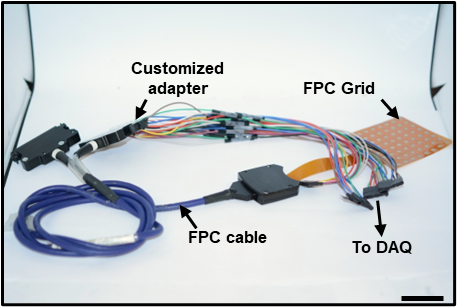
**

**Fig. S8.** Custom connection scheme to capture data from the FPC grid. The customized adapter was custom-made so that the grid could be used with a Intan Recording Controller and amplifier. Scale bar = 5 cm.

**
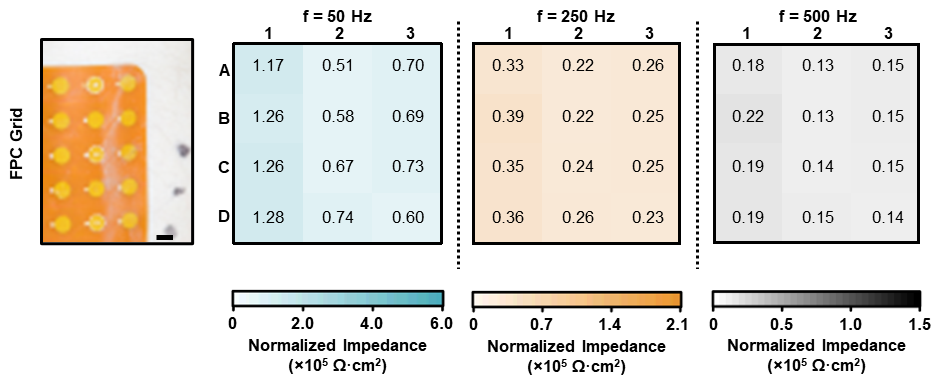
**

**Fig. S9.** Normalized skin-electrode impedance from a subset of electrodes in the FPC grid. Scale bar = 5 mm.

**
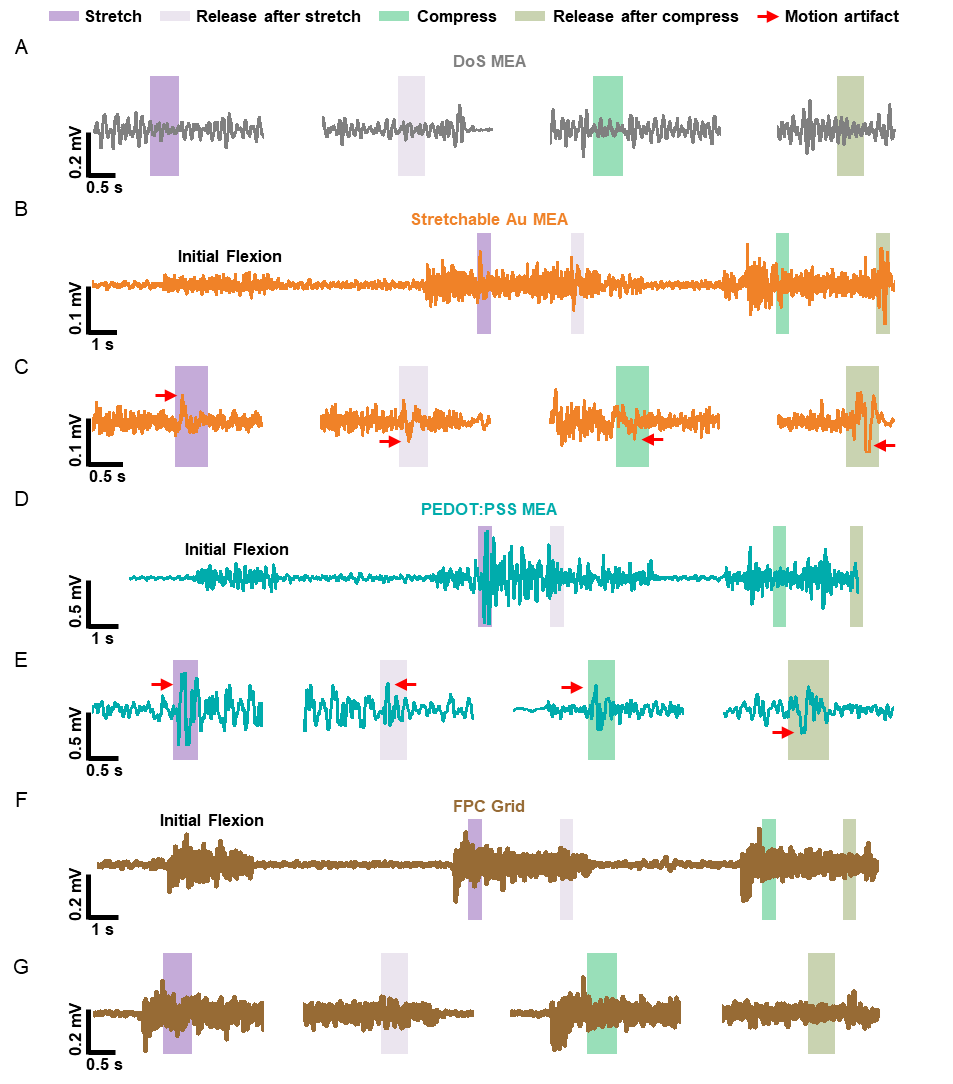
**

**Fig. S10.** Effect of skin deformation-induced motion on DoS and wearable MEAs. (*A*) Zoomed-in view of EMG data around the duration of skin deformation recorded with the DoS MEA. (B) EMG data recorded with three flexions of the flexor group of muscles in the forearm using the stretchable Au MEA. The initial flexion was done without any skin deformation to the MEA. The following two flexions were performed with skin deformation, first stretching the skin around the edge of the stretchable Au MEA and then compressing the skin. (*C*) Zoomed-in view of EMG data around the duration of skin deformation recorded with the stretchable Au MEA. (*D*) EMG data recorded with three flexions of the flexor group of muscles in the forearm using the printed PEDOT:PSS MEA. The initial flexion was done without any skin deformation to the MEA. The following two flexions were performed with skin deformation, first stretching the skin around the edge of the DoS MEA and then compressing the skin. (*E*) Zoomed-in view of EMG data around the duration of skin deformation recorded with the printed PEDOT:PSS MEA. (*F*) EMG data recorded with three flexions of the flexor group of muscles in the forearm using the FPC Grid. The initial flexion was done without any skin deformation to the MEA. The following two flexions were performed with skin deformation, first stretching the skin around the edge of the FPC Grid and then compressing the skin. (*G*) Zoomed-in view of EMG data around the duration of skin deformation recorded with the FPC Grid.

**
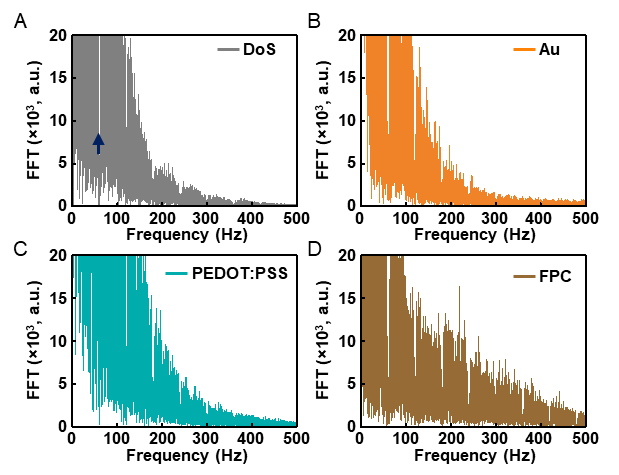
**

**Fig. S11**. Fast Fourier Transform data from each of the arrays. Each graph shows the FFT data from the EMG signal during the initial contraction of the skin-deformation induced motion artifacts comparison from a single subject and (*A*) is the average data from all channels recorded with the DoS MEA, and the blue arrow marks the effect of the notch filter; (*B*) is the average data from all channels recorded with the stretchable Au MEA; (*C*) is the average data from all channels recorded with the PEDOT:PSS MEA; and (*D*) is the average data from all channels recorded with the FPC grid.

**
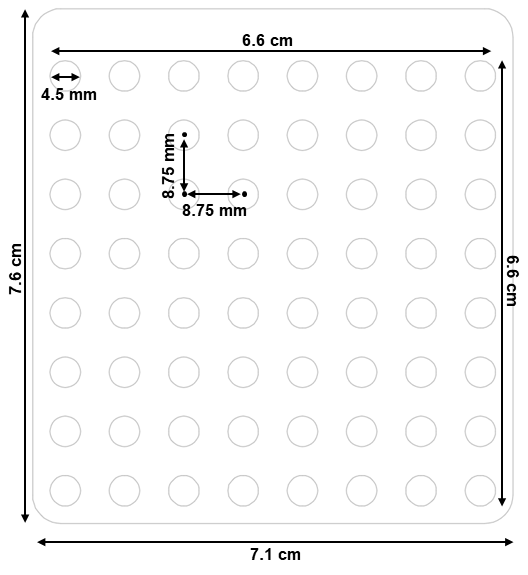
**

**Fig. S12.** Detailed geometrical dimensions of the FPC grid.

**
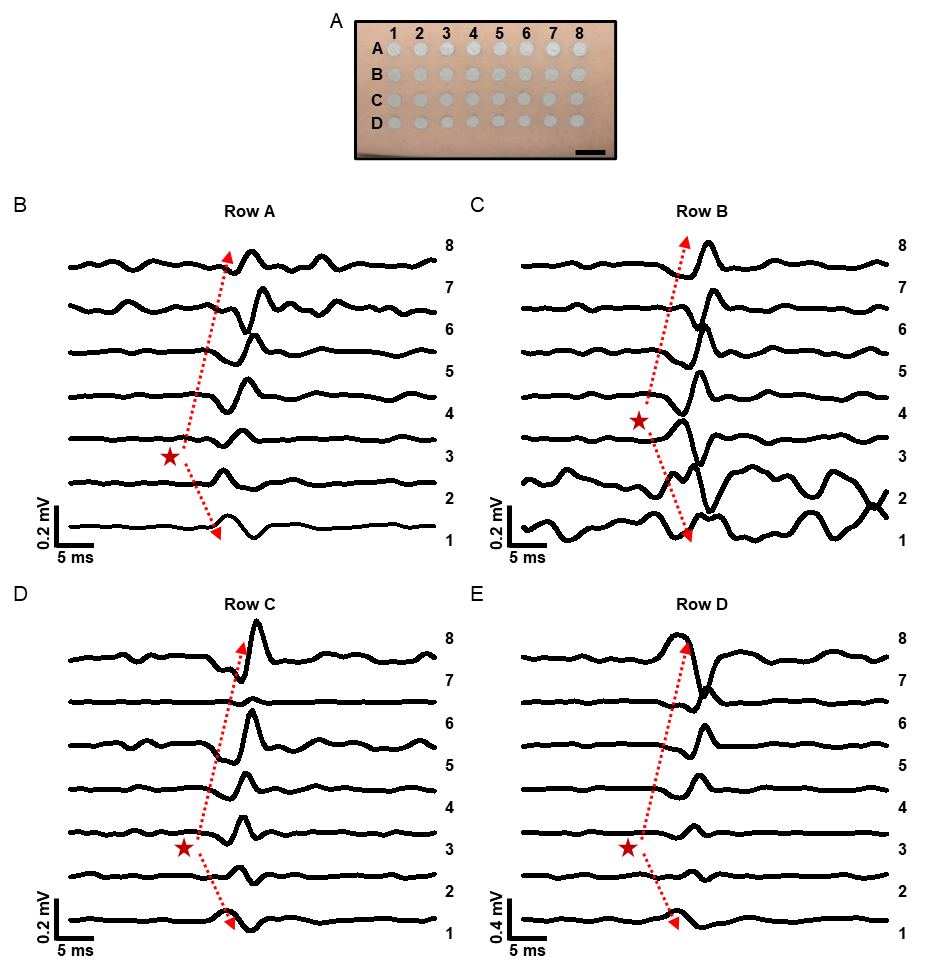
**

**Fig. S13.** Propagation maps for each row from the high-density DoS MEA. (*A*) Layout of the DoS MEA with the rows labeled with letters and the columns labeled with numbers. Note that the propagation maps were constructed using a bipolar method, where the difference of the neighboring electrodes in one row was used to find motor units instead of using the individual data channel data. Scale bar = 1 cm. (*B*) Propagation map of row ‘A’ of the high-density DoS MEA. The change in the inflection of the wave in the third trace from the bottom (indicated by the red star) denotes the innervation zone and the red arrows indicate the characteristic ‘V’ pattern indicating propagation of the motor unit action potential in different directions from the innervation zone. (*C*) Propagation map of row ‘B’ of the high-density DoS MEA. (*D*) Propagation map of row ‘C’ of the high-density DoS MEA. (*E*) Propagation map of row ‘D’ of the high-density DoS MEA.


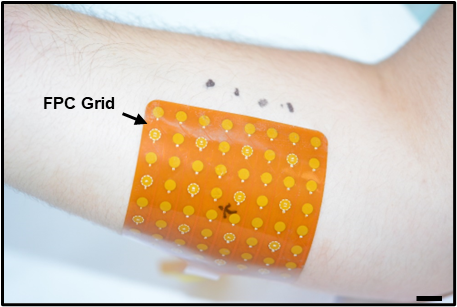


**Fig. S14.** Placement of FPC grid above the forearm flexors for the motor unit detection comparison. Scale bar = 1 cm.

**
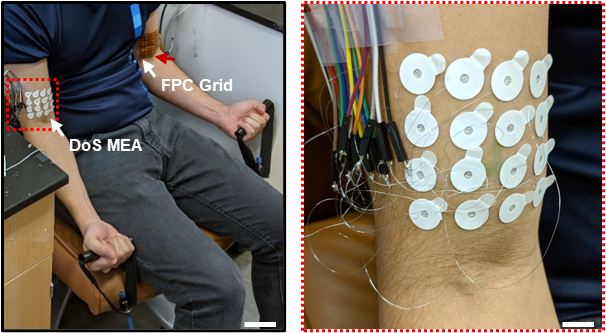
**

**Fig. S15.** Image of the setup for evaluating the quality of the EMG signals during substantial muscle movement underneath the skin. The image on the left shows the DoS MEA on the right arm of the subject and the FPC grid on the left arm. Over several repetitions of the exercise, the grid started to delaminate (indicated by the red arrow, scale bar = 10 cm). The image on the right (scale bar = 1 cm) shows the connection of the DoS MEA to the data acquisition system using the tape to connect stainless-steel wires directly to the DoS electrodes.

**
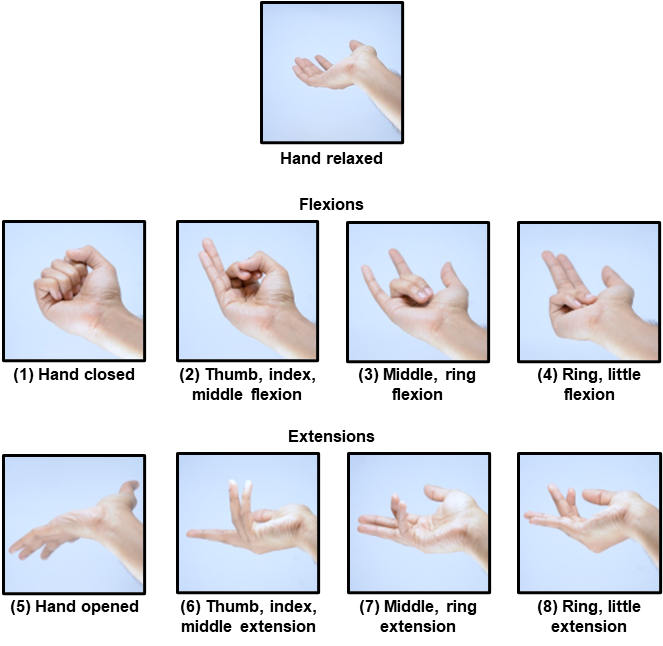
**

**Fig. S16.** Various finger gestures performed throughout this work for multiple experiments.

**
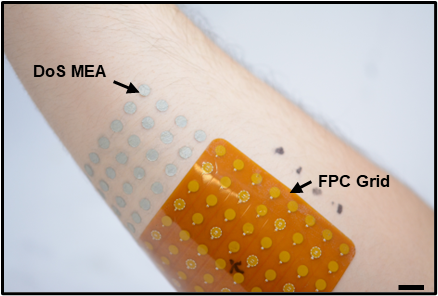
**

**Fig. S17.** Reconfigured DoS MEA used in conjunction with the FPC grid. The image above is of arrangement 3, with all the DoS electrodes positioned on one side of the FPC grid. Scale bar = 1 cm.

**
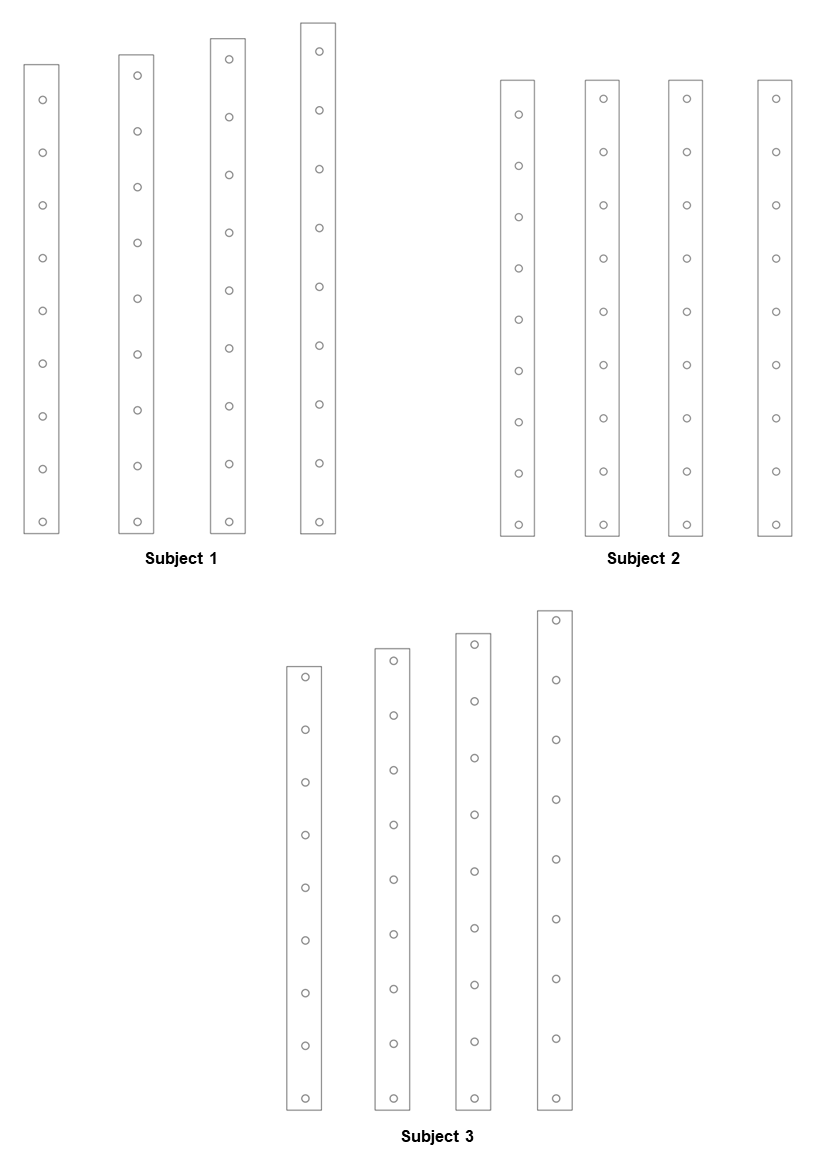
**

**Fig. S18.** Custom stencils for each subject for the finger gesture classification experiment. Each subject has a unique circumference of their forearm and the stencils are linear arrays placed circumferentially on 4 positions of the forearm, each position spaced ~2 cm apart.

**
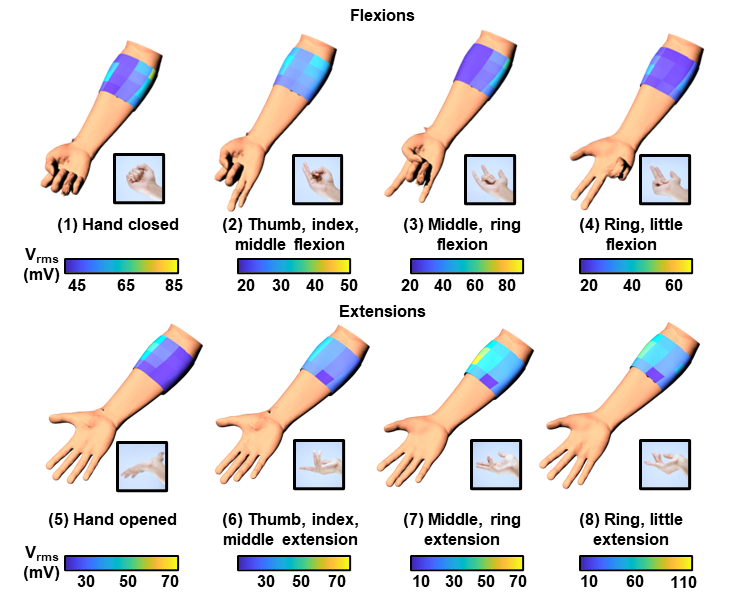
**

**Fig. S19.** Medial view of forearm excitation maps for each finger gesture. The top row shows the V_rms_ maps from the flexion gestures and the bottom row shows the V_rms_ maps from the extension gestures.

**
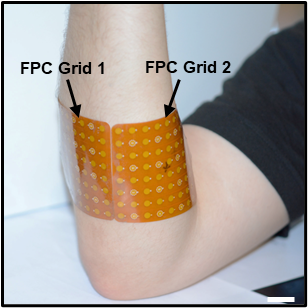
**

**Fig. S20.** FPC grids placement around the forearm for the finger gesture classification experiment. Scale bar = 2 cm.

**
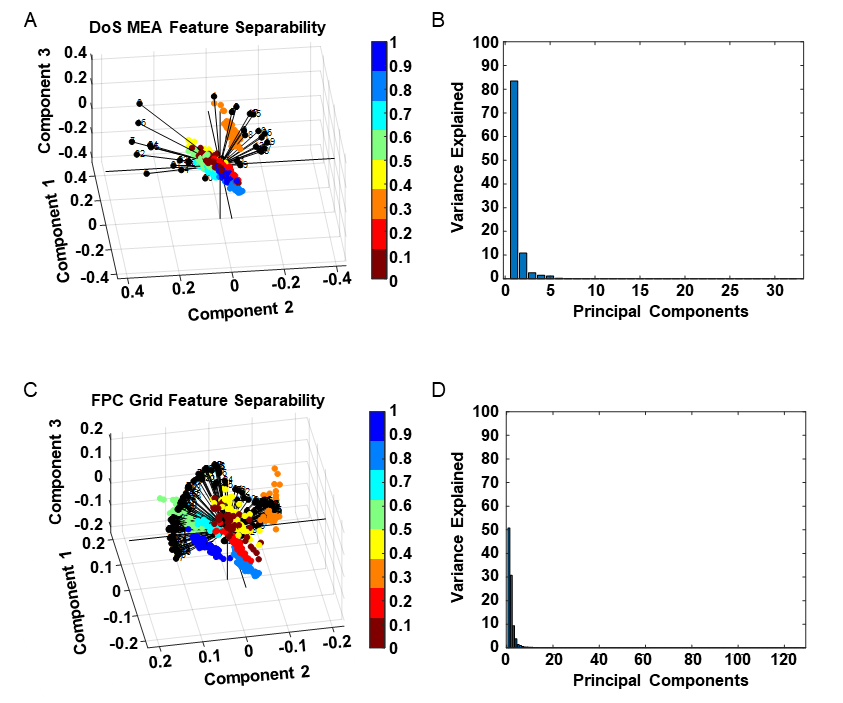
**

**Fig. S21.** Principal component analysis of EMG features. (*A*) Principal component map with the first three components obtained from data recorded with the customized DoS MEA. The colors in the color bar, from bottom to top represent gestures 1-8 which correspond to (1) Hand closed; (2) Thumb, index, middle flexion; (3) Middle, ring flexion; (4) Ring, little flexion; (5) Hand opened; (6) Thumb, index, middle extension; (7) Middle, ring extension; and (8) Ring, little extension. (*B*) Percentage of variance based on each principal component identified from data recorded with the customized DoS MEA. (*C*) Principal component map with the first three components obtained from data recorded with the two FPC grids. (*D*) Percentage of variance based on each principal component identified from data recorded with the two FPC grids.

**References**

1. Bihar E, Roberts T, Zhang Y, Ismailova E, Hervé T, Malliaras GG, De Graaf JB, Inal S, Saadaoui M (2018) Fully printed all-polymer tattoo/textile electronics for electromyography. *Flexible and Printed Electronics* 3:034004.

2. Kwon YT, Norton JJS, Cutrone A, Lim HR, Kwon S, Choi JJ, Kim HS, Jang YC, Wolpaw JR, Yeo WH (2020) Breathable, large-area epidermal electronic systems for recording electromyographic activity during operant conditioning of H-reflex. *Biosens Bioelectron* 165:112404.

3. Dai C, Hu X (2019) Extracting and Classifying Spatial Muscle Activation Patterns in Forearm Flexor Muscles Using High-Density Electromyogram Recordings. *Int J Neural Syst* 29:1850025.
